# Development of a LC–MS/MS Method for the Simultaneous Determination of the Mycotoxins Deoxynivalenol (DON) and Zearalenone (ZEA) in Soil Matrix

**DOI:** 10.3390/toxins13070470

**Published:** 2021-07-07

**Authors:** Arne Kappenberg, Lena Marie Juraschek

**Affiliations:** Institute of Crop Science and Resource Conservation (INRES), Soil Science and Soil Ecology, University of Bonn, Nussallee 13, 53115 Bonn, Germany; lena.juraschek@uni-bonn.de

**Keywords:** mycotoxins, soil texture, LC–MS/MS, deoxynivalenol (DON), zearalenone (ZEA)

## Abstract

Mycotoxins, toxins of fungal origin, can directly or indirectly contaminate food and feed and are poisonous to livestock and humans. While a large amount is known about their occurrence in crops, food, and feeds, little is known about mycotoxin amounts in soil. However, soil is known as a major fungal habitat and a potential sink for mycotoxins in the environment. Furthermore, there is neither a reliable detection nor an extraction method for mycotoxins testing in different soil textures or for potential deficits due to aging processes. Therefore, the aim of the present study was to present a reliable extraction and detection method for the simultaneous quantification of the most common mycotoxins, deoxynivalenol (DON) and zearalenone (ZEA), via liquid chromatography-tandem mass spectrometry (LC–MS/MS). This method was validated with six different samples with different textures and different soil organic matter (SOM). Deuterated standards were used to overcome possible matrix effects. This extraction method could eliminate potential aging processes. The recovery rate was always >80% for DON and >82% for ZEA. The quantification limits were 1 ng per g soil for DON and 0.5 ng per g soil for ZEA.

## 1. Introduction

Mycotoxins, secondary metabolites of the soil fungal genera *Aspergillus*, *Fusarium*, and *Penicillium* [1], can cause a variety of adverse health effects and pose a serious health threat to humans and livestock [2]. They also have the potential to induce both acute and chronic health effects via ingestion, skin contact, inhalation, and entering the blood stream and lymphatic system [2]. The adverse health effects of mycotoxins range from acute poisoning to long-term effects, such as immune deficiency and cancer [1,2,3]. Recent research associated mycotoxin exposure with human hepatocellular carcinomas [4]. It is assumed that 250,000 deaths per year from hepatocellular carcinomas in China and Sub-Saharan Africa could be caused by the daily intake of >1.4 μg mycotoxins [1,4]. Such toxins can form and accumulate on crop residues and, therefore, also in soil, both prior to and after farming, as well as directly on the crops during harvest and their subsequent drying and storage [2,5]. Contamination of food and feedstuffs is unavoidable and unpredictable, resulting in an estimated loss of 25% of the worldwide crop yield per year [3,6,7]. Additionally, global warming—and the associated hot, dry summers and humid winters and springs—has led to an increase in worldwide fungal infestations and thus mycotoxin occurrence [8,9,10].

The most common and most prominent mycotoxins in soil are deoxynivalenol (DON) and zearalenone (ZEA), produced by *Fusarium*, and are commonly spread contaminants in animal feed, mostly in cereals and forages [11]. Furthermore, DON and ZEA are stable under varying environmental conditions, causing a diversity of toxic effects in humans, farm animals, and laboratory animals [11,12]. DON, also known as vomitoxin [2,13,14], is directly transported to the brain after consumption, where it disturbs and destroys dopaminergic receptors [15,16]. DON is also able to resist high temperatures and can even be transferred from the animal to its products and, therefore, is present even in heat-treated products, such as milk and cheese [17]. The LD50 value for mice ranges from 46 to 78 mg/kg (oral DON administration) [18]. Compared to DON, the adverse health effects due to ZEA contamination/exposure are mainly shown by its chronic effects [19]. The toxic properties result from its estrogenic characteristics due to its identical structural shape with naturally occurring estrogens. ZEA is also known as an F-2 toxin, causing, for example, vulvovaginitis and estrogenic responses [20,21]. ZEA occurs in many plants and their products, including corn, barley, fruits and vegetables, moldy hay, and in pelleted feed rations [7,19,20]. Although the compound is not principally toxic, concentrations of 1–5 ppm are already sufficient to cause physiological response reactions. The International Agency of Research on Cancer (IARC) have not been clearly confirmed carcinogenic properties for ZEA [7,22]. However, as DON and ZEA are the most common contaminant of grains, grain-based products, fruits, and vegetables, their occurrences in food and feed frequently exceed 90% of the total number of samples [16], which means they are the best possible marker toxins for determining the general occurrence of mycotoxins.

Current monitoring has only focused on mycotoxins in grains and crops [5,7,8,9,10,19,20,23,24]. However, it is known that the contamination of agricultural soil with mycotoxins plays a significant role in the contamination of grains and crops [25,26,27,28]. Mortensen et al. [25,26] analyzed ochratoxin A (OTA) and ZEA in spiked soil samples using high-pressure liquid chromatography (HPLC) with fluorescence detection after extraction (shaking for 30 min.) with methanol/water (9:1, *v/v*) and solid-phase extraction (SPE) and found rapid dissipation of both compounds; however, the studied concentration range (0.1–1 ng per g soil) was likely to be far above natural contamination levels. The detections limits were 0.1 ng/g for OTA and 1.0 ng/g for ZEA. Furthermore, Muñoz et al. [27] successfully analyzed DON in Luvisols in plastic mulching practices via liquid chromatography coupled with high-resolution mass spectrometry (LC-HRMS). For ultrasonic extraction, they used methanol/water (9:1, *v/v*). They were able to detect concentrations of DON in a range from LOD to 32.1 ng per g soil (LOD 1.1 ng/g). However, they were only able to quantify DON and not ZEA. Hartman et al [28,29] analyzed ZEA in topsoil (0–10 cm) through Soxhlet extraction with pure methanol for 18 h and found up to 3.8 ng ZEA per g soil (LOQ 0.7 ng/g). Furthermore, Gromadzka et al. [30] investigated the migration of ZEA in the agricultural environment and therefore examined ZEA contents by means of HPLC with fluorescence detection in wheat, corn, water, and soil. The soil samples were extracted with acetonitrile/water (90:10, *v/v*) after grinding. They were able to confirm the presence of ZEA in all of the soil samples over a period of three years, with an average content ranging from 2.0 ± 0.7 to 25.5 ± 9.7 ng ZEA per g soil (LOD 0.5 ng/g). However, we are not aware of any method that has ever been tested for use with different soil textures, where a broader range of mineral and particularly soil organic matter (SOM) constituents [31] may interfere with the analytes, thus requiring specific methodological adaptations for analyses, similar to those used for pesticides [32], antibiotics [33], or polychlorinated aromatic hydrocarbons [34]. Consequently, no studies have analyzed DON and ZEA simultaneously in soil and/or validated them against different soil textures and SOM, nor against aging processes; that is, the increase in the binding strength of mycotoxins with increasing contact time in soil.

However, for the determination and quantification of mycotoxins in complex matrices, such as cereals and plasma, liquid chromatography–tandem mass spectrometry (LC–MS/MS) has been used extensively and has been shown to be the best [8,24,35,36,37]. With the aim of reducing the matrix effects by reducing the interference from the extraction step, a wide range of solvents and extraction methods were tested [8,24,35,36,37]. Mycotoxins are mostly soluble in polar and slightly polar solvents, and insoluble in apolar solvents [38,39]. Thus, polar solvents and mixtures of polar solvents, such as acetone, acetonitrile, chloroform, dichloromethane, ethyl acetate, or methanol with small amounts of acids or water are commonly used [38,39]. De Santis et al. [24] validated a multi-mycotoxin and multi-matrix method in liquid chromatography–tandem mass spectrometry (LC–MS/MS) with the purpose of overcoming specific matrix effects and analyzing complex cereal-based samples within the Italian Total Diet Study project. However, because that was a diet study, soil was not tested. Thus, it is still not clear which method is best for the determination of DON and ZEA in complex and different soil matrices.

The present study aims to develop a method for the simultaneous extraction and determination of DON and ZEA in a complex soil matrix. The method was validated with six different soils of different textures and SOM contents. This study was the first to test whether this method can eliminate possible binding processes due to aging.

## 2. Results and Discussion

### 2.1. LC–MS/MS Optimization

For hte optimization of LC–MS/MS parameters, tuning solutions of DON (100 ng/mL) and ZEA (100 ng/mL) were applied directly, and mycotoxin determination was performed in positive ionization mode. Formic acid (0.1%) and ammonium formate (2 mmol) were added to facilitate the formation of the protonated precursor ion or ammonium adduct (see also De Santis et al., [24]). Whereas the two most intense product ions were used for the identification of the mycotoxins DON and ZEA, we only used the most intense product ion (quantifier) for the quantification in soil samples. For the identification of the internal standards (^13^C_15_-DON, ^13^C_18_-ZEA), we only used the quantifier. Specific MS/MS parameters are presented in Table 1.

### 2.2. Extraction Steps 

Many extraction methods and different solvents have been tested to reduce the matrix effects by reducing the interference from the extraction step in the determination of mycotoxins; however, these were mostly tested in grains, crops and food [24,35,36,37]. As DON and ZEA are only soluble in polar solvents [39], we tested common extraction methods with mixtures of polar organic solvents [24,25,26]. Therefore, we tested: (i) ultrasonic bath, (ii) shaking, (iii) accelerated solvent extraction (ASE 350, Dionex by Thermo Fisher Scientific, Waltham, MA, United States), and (iv) pressurized solvent extraction (Speed extractor E-914, Büchi Labortechnik AG, Flawil, Switzerland) with commonly used solvents: (i) acetonitrile, (ii) water/acetonitrile (80:20), (iii) methanol/water (90:10), and (iv) acetonitrile/water/glacial acetic acid (79:20:1). Extraction was tested with soils with either high amounts of clay (69%, Ascheberg), sand (96%, Auweihler), or silt (75%, Berrenrath), and one soil sample containing all of the aforementioned soil textures in equal amounts (silty clay loam, Frankenforst, Table 2). Furthermore, to test whether soil organic carbon (SOC) has an influence on extraction efficiency, we tested two topsoils with high amounts of SOC (Berrenrath and Frankenforst) and two subsoils with low amounts of SOC (Auweihler and Ascheberg, Table 2).

Accelerated and pressurized solvent extraction (ASE and Büchi) enabled the worst results. Regardless of which solvents were used, the recovery using ASE was between 26 and 35% for DON and 24 and 43% for ZEA. For Büchi, recovery was between 27 and 38% for DON and 23 and 43% for ZEA (Table 3). However, the best recovery rates were enabled by the comparatively simple extraction methods: (i) shaking (DON: 46–69%; ZEA: 42–71%) and (ii) mainly ultrasonic bath (DON: 56–85%; ZEA: 51–85%). This was probably because the soil texture is additionally affected by mechanical stress through shaking or ultrasonic treatment. The most efficient solvent mixture among all of the tested extraction methods was acetonitrile/water/glacial acetic acid (79:20:1) (Table 3). Overall, we recommend the use of acetonitrile/water/glacial acetic acid (79:20:1) via ultrasonic treatment for the extraction of DON and ZEA in soil. The recovery rates for this treatment were always >80% for ZEA and >82% for DON. The mechanical stress of the ultrasonic bath and the acidified extraction solution probably improved the efficiency of the extraction, as both the ultrasonic treatment and the acidified extraction solution are able to break up the interactions between the toxins and other sample components such as proteins or sugars [38,39].

### 2.3. Method Performance

#### 2.3.1. Linearity

The linearity of DON and ZEA was tested by means of the evaluation of the determination coefficients (R^2^) and *p*-values (***) with a six-point calibration in neat solvent mixture (A/B, 50:50) and in spiked samples with different soil textures (Auweihler 96% sand; Ascheberg 69% clay; Berrenrath 75% silt; and Frankenforst mixture) (Table 4). The results confirm the high quality of the method with R^2^ > 0.9885 and *p*-values < 0.0001 for all of the soil samples investigated (Table 4). Thus, linearity could be confirmed for all soil samples and soil textures.

#### 2.3.2. Recovery and Matrix Effect

Although we recommend using ^13^C_15_-DON and ^13^C_18_-ZEA as internal standards to compensate for possible matrix effects, we evaluated the following parameters according to De Santis et al. [24] without a deuterated internal standard to verify the quality of our method. Therefore, we calculated the recovery and matrix effect by means of the the evaluation of the apparent recovery (R_A_), signal suppression/enhancement (SSE), extraction recovery (R_E_), and the relative standard deviation of repeatability of R_A_ for DON and ZEA in sand, clay, silt, and in silty clam loam (mix). The results (obtained from calibration curves) are presented as apparent recovery (R_A_), signal suppression/enhancement (SSE), extraction recovery (R_E_), and the relative standard derivation (RSD_r_) calculated for R_A_ (Table 5). We could not see any significant matrix interference. R_A_ and SSE are always >81%. The differences between R_A_ and SSE are always <5%, and the RSD_r_ is always <10%. The results demonstrate the suitability of our method for the extraction and quantification of DON and ZEA in validated soil matrices.

#### 2.3.3. Aging

Recovery was tested with spiked soil samples from Ascheberg (69% clay), Auweihler (96% sand), Berrenrath (75% silt), and Frankenforst (mixture) immediately (0 days) and again to test aging processes—that is, the increase in mycotoxin binding strength with increasing contact time in soil—after 28 days (Table 6). The recovery rate was found to be >82% for all of the soil samples and was not dependent on the soil texture or SOM. The results show that possible adsorption or aging processes due to the extraction method can be eliminated. The results are almost equally independent of the duration of storage (0/28 days) and the temperature conditions (8/21 °C) during storage.

#### 2.3.4. Routine Limit of Detection and Quantification

The sensitivity of the analytical method using LC–MS/MS was determined by a limit of detection (LOD) and limit of quantification (LOQ). These were calculated as signal-to-noise (S/N) ratios of 3 and 10, respectively. The LOD of the analytical method for DON was 0.3 ng per g soil and for ZEA 0.15 ng per g soil. The LOQs were 1 for DON and 0.5 ng per g soil for ZEA.

#### 2.3.5. Validation at Natural Concentration Levels

**Figure 1:**
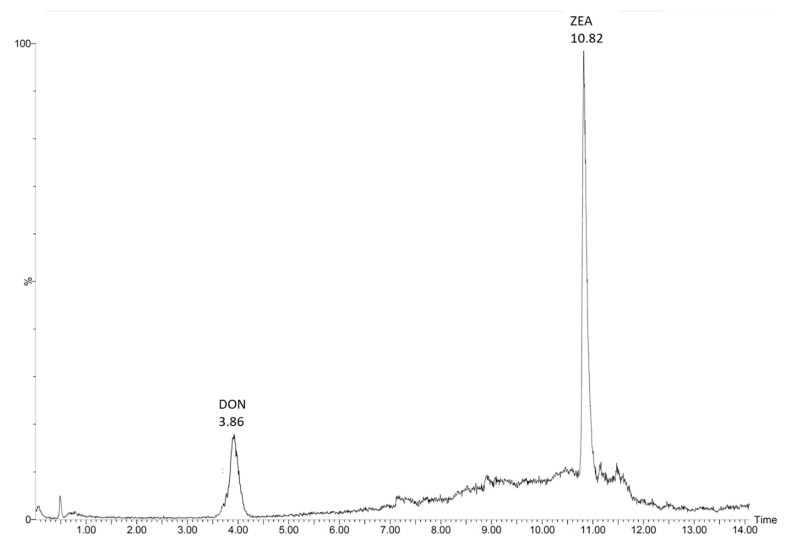
LC–MS/MS chromatogram of deoxynivalenol (DON) and zearalenone (ZEA) from a sample location in Rackwitz, Germany, without use of fungicides and no-till cultivation over the last 20 years. Mycotoxin concentrations shown represent natural levels (5.85 ± 0.34 ng DON and 1.91 ± 0.15 ng ZEA per g soil) without prior spiking. LOQs are 1.0 und 0.5 ng/g for DON and ZEA, respectively.

To validate the extraction method, soil samples with natural levels of mycotoxins were extracted and quantified for DON and ZEA (Figure 1) using ^13^C_15_-DON and ^13^C_18_-ZEA as internal standards. Both sites are characterized by no plowing and no fungicide use over the last 20 years. To test aging processes, samples from Lüttewitz and Rackwitz were analyzed both directly after sampling and 28 days after storage at 8 °C and 21 °C. Here, no differences due to the influence of storage duration and storage temperature could be detected. The concentrations of DON and ZEA in all samples, whether analyzed immediately or after 28 days (storage at 8 °C and 21 °C), were 9.16 ± 0.46 ng per g soil and 1.07 ± 0.06 ng per g soil in the samples from Lüttewitz and 5.85 ± 0.34 ng per g soil and 1.91 ± 0.15 ng per g soil in the samples from Rackwitz for DON and ZEA, respectively. The recovery was > 86%, and the relative standard deviation <6% for all of the soil samples tested. It is difficult to make comparisons with other studies because hardly any studies have analyzed DON and ZEA in soils. Muñoz et al. [27] analyzed DON in Luvisol in plastic mulching practices and found that amounts of DON ranged from LOD to 32.1 ng per g of soil (LOD 1.1 ng/g) to 32.1 ng per g soil. In non-covered soil, they analyzed a DON concentration of 9.1 ± 7.9 ng per g soil. Hartman et al. [28,29] and Gromadzka et al. [30] analyzed ZEA in topsoil and found amounts between 2.0 ± 0.7 and 25.5 ± 9.7 ng per g soil, respectively. The results show that our results are similar to those of Muñoz et al [27], Hartman et al. [28,29], and Gromadzka et al. [30]. Our results clearly show that the described method can be used for soil samples with natural contents of DON and ZEA. In addition, possible aging processes such as increased binding of mycotoxins to the soil matrix could be effectively eliminated using the extraction method.

## 3. Conclusions

Here we present a reliable extraction and LC–MS/MS method for the simultaneous determination of DON and ZEA in soil. We adapted existing methods for the determination of toxins in food and feed for the determination of DON and ZEA in different soil textures. The method was validated with six different soils with varying soil textures and was tested for detection and quantification limits, apparent and extraction recovery, signal suppression/enhancement, linearity, and binding processes due to different storage time and conditions. Sample selection included four sampling sites without natural amounts of DON and ZEA and two with natural amounts of DON and ZEA. Samples without natural amounts of mycotoxins were spiked with DON and ZEA. The internal standards ^13^C_15_-DON and ^13^C_18_-ZEA were added to establish the analytical method matrix-independent. The best results were obtained through the use of acetonitrile/water/glacial acetic acid (79:20:1) via ultrasonic extraction. All samples passed linearity (DON: R^2^ > 0.9885; *p* < 0.0001; ZEA: R^2^ > 0.9886; *p* < 0.0001), recovery (DON > 80%; ZEA > 82%), and an aging test. LOQ was 1 ng per g soil for DON and 0.5 ng per g soil for ZEA. Thus, the present results indicate that the method is most suitable for all soil samples and textures. With satisfactory accuracy, recovery, LOQ, and linearity, we can confirm the very good performance of our method. Furthermore, it can be shown that our method can exclude potential adsorption processes due to aging.

## 4. Materials and Methods

### 4.1. Chemicals and Reagents

For extraction—or/and LC mobile phase solutions, methanol (MeOH, hypergrade for LC–MS, Merck KGaA, Darmstadt, Germany), acetonitrile (hyper grade for LC–MS, Merck KGaA, Darmstadt, Germany), formic acid (p.a., ACS, Carl Roth GmbH + Co. KG, Karlsruhe, Germany), glacial acetic acid (ACS, Reag. Ph. Eur., VWR chemicals, Fontenay- sous-Bois, France), ultra-pure water, and ammonium formate (for mass spectrometry, ≥99.0, Sigma-Aldrich, St. Louis, MO, USA) were used. Ultra-pure water (water) was produced using a Milli-Q system (Millipore, Bedford, MA, USA). For filtering, glass fiber GF6 filters (Whatman, Sigma-Aldrich, St. Louis, MO, USA) were used. The certified standard solutions of DON and ZEA and ^13^C_15_- DON and ^13^C_18_- ZEA were purchased from LGC Standards GmbH (Wesel, Germany).

Solvents for the separation of mycotoxins (mobile phase) consisted of (A) water and (B) methanol, both buffered with 2 mmol per L ammonium formate and 0.1% formic acid. The DON and ZEA stock solutions (0.1, 0.5, 1, 5, 10, 20, 30, 50, 75, 100, and 150 ng per mL) were prepared by dissolving appropriate volumes of each compound in a mixture of solvents A and B (50:50). As an internal standard, ^13^C_15_- DON (10 ng per mL) and ^13^C_18_- ZEA (20 ng/mL) were used. All solutions were stored at −20 °C in amber glass vials and darkness until use.

### 4.2. Soil Samples

Overall, six different soils were tested, and these soil samples covered a wide range of soil textures, containing either high amounts of clay (69%, Ascheberg), sand (96%, Auweihler), silt (75%, Berrenrath, 85% Lüttewitz), or a mixture of all of the aforementioned textures (silty clam loam, Frankenforst, and Rackwitz) and soil organic matter content (SOC) (Table 1). Samples were collected with a sampling ring at a depth of 5 cm (topsoil) and/or at a depth of about 25 cm (subsoil). After collection, the samples were frozen at −20 °C. Soil samples from the Ascheberg, Auweihler, Berrenrath, and Frankenforst sites were expected to contain no natural amounts of DON and ZEA, while natural levels of DON and ZEA were expected in Lüttewitz and Rackwitz, as no fungicides have been applied there, nor has any plowing been done in the last 20 years.

### 4.3. Testing of Different Extraction Methods and Solvents

Different extraction methods using mixtures of polar organic solvents were tested using soil samples from four different sites in Germany, with either a high proportion of clay (69%, Ascheberg), sand (96%, Auweihler), or silt (75%, Berrenrath) as well as with a soil sample containing all soil textures in equal proportions (silty clay loam, Frankenforst). To test whether soil organic carbon (SOC) had an effect on the extraction efficiency of the results, two topsoils with high SOC content (Berrenrath and Frankenforst) and two subsoils with low SOC content (Auweihler and Ascheberg) were tested (Table 2). The tested extraction methods were: (i) ultrasonic bath, (ii) shaking, (iii) accelerated solvent extraction (ASE 350, Dionex by Thermo Fisher Scientific, Waltham, MA, United States), and (iv) solvent extraction under pressure (Speed extractor E-914, Büchi Labortechnik GmbHAG, Flawil, Switzerland) with the following solvents: (i) acetonitrile, (ii) water/acetonitrile (80:20, *v/v*), (iii) methanol/water (90:10, *v/v*), and (iv) acetonitrile/water/glacial acetic acid (79:20:1, *v/v/v*). Six replicates were set up for each extraction method and solvent, and all possible combinations of extraction method and solvent were tested (Table 3). An ultrasonic bath with acetonitrile/water/glacial acetic acid (79:20:1, *v/v/v*) was finally selected for soil sample preparation and extraction (see Section 2.2 and Section 4.4).

### 4.4. Soil Preparation and Extraction Method 

Samples were sieved (2 mm) before analysis to homogenize and remove plant residues. Subsequently, 20 g of freeze-dried samples were weighed into 100 mL centrifuge tubes and spiked with 20 µL of DON (10 ng per g soil) and 10 µL of ZEA (5 ng per g soil) (excluding sites with natural levels of DON and ZEA, Table 2). Soil samples were then extracted once with 25 mL of a mixture water/acetonitrile/glacial acetic acid (79:20:1, *v/v/v*) for 60 min. in an ultrasonic bath. After centrifugation for 5 min at 3500 rpm, the extract was filtered into a vacuum pump using a GF6 glass fiber filter (Whatman, Sigma-Aldrich, St. Louis, MO, USA). For better sample purification, the glass filter was used in a double layer (tested beforehand). Samples were completely evaporated using the SyncorePlus (Büchi Labortechnik AG, Flawil, Switzerland) and filled up with a 1 mL injection solution mixture A/B (50:50). Furthermore, the internal standards ^13^C_15_-DON and ^13^C_18_-ZEA were added before extraction. The use of ^13^C_15_-DON and ^13^C_18_-ZEA as internal standards was essential to demonstrate the matrix independence of the analytical method since the internal standard compensates for the losses of the target analytes during extraction and ion suppressions during ionization [24].

### 4.5. Chromatographic Separation and Quantification

Liquid chromatography was performed on a Finnigan Surveyor 1100 HPLC system (Thermo Fisher Scientific, Waltham, MA, United States). An injection volume of 10 µl was used and separated on a Kinetex 2.6 µm Biphenyl 100 Å 3 mm i.d. × 50 mm column (Phenomenex, Torrance, CA, USA) at 40 °C using a constant flow of 0.3 mL per min. The mobile phase consisted of a gradient of water (mobile phase A) and MeOH (mobile phase B), both containing 2 mmol/L ammonium formate and 0.1% (*v/v*) formic acid. The gradient was as follows: initial 95% A (5% B), linear decrease to 12 min 5% A (95% B), and hold for 2 min and set to the initial gradient.

The LC was coupled to a triple-quadrupole mass spectrometer (TSQ Quantum Ultra, Thermo Fisher Scientific, Waltham, MA, USA). A heated electrospray ionization (HESI) source operated in positive ionization mode (HESI+). Nitrogen was used as a collision gas, and the MS operated in selected reaction monitoring SRM mode. Collision energy was optimized for each precursor ion and product ion (Table 2). The limit of quantification (LOQ) was 1 ng DON per g soil and 0.5 ng ZEA per g soil.

### 4.6. Method Performance

The limits of detection (LODs) were calculated based on a signal-to-noise (S/N) ratio of 3:1 and the limits of quantification (LOQs) at a S/N ratio of 6:1 by injecting pure solvent standard solutions at various concentrations. Verification and validation were performed on different analyzed matrices. In order to test and exclude the influence of matrix effects on the levels of DON and ZEA, ^13^C_15_-DON and ^13^C_18_-ZEA were spiked into all soil samples containing natural amounts of DON and ZEA (Rackwitz and Lüttewitz) before extraction. For method performance, all soil samples were processed in replicates (n = 8).

#### 4.6.1. Linearity

Linearity was tested by preparing an internal calibration curve with six concentration levels for DON and ZEA, with and without ^13^C_15_-DON and ^13^C_18_-ZEA. Calibration was repeated six times (n = 6), and the mean values from the replicates were used. The linearity of the mycotoxins was tested in neat solvent mixture A/B (50:50) and in spiked soil samples (before extraction) without natural amounts of mycotoxins from Auweihler (sand), Ascheberg (clay), Berrenrath (silt), and Frankenforst (silty shell loam) (Table 1).

#### 4.6.2. Recovery and Matrix Effect

The use of ^13^C_15_-DON and ^13^C_18_-ZEA as internal standards was essential for demonstrating the matrix independence of the analytical method since the internal standard compensates for the losses of target analytes during extraction and ion suppression during ionization [24]. However, here we evaluated the following parameters according to De Santis et al. [24] without deuterated internal standard to verify the quality of our method. Apparent recovery (R*_A_*), signal suppression/enhancement (SSE), and extraction recovery (R*_E_*) were calculated according to De Santis et al. [24]. Values were calculated using a six-point calibration curve with the ranges 1–100 ng/mL and 0.5–100 ng/mL for DON and ZEA, respectively (Table 4). To calculate R*_A_*, SSE, and R*_E_*, we used the following equations according to De Santis et al. [24]:

(1) $$R_{A}\;\left(\%\right)=100\times \frac{slope_{spiked\;sample}\;}{\begin{array}{c} slope_{neat\;solvent} \end{array}}$$

(2) $$\mathrm{SSE}\;\left(\%\right)=100\times \frac{slope_{spiked\;extract}\;}{\begin{array}{c} slope_{neat\;solvent} \end{array}}$$

(3) $$R_{E}\;\;\left(\%\right)=100\times \frac{R_{A}\;}{\begin{array}{c} SSE \end{array}}$$

#### 4.6.3. Aging

The water holding capacity (WHC) and the initial water content of the soil samples were determined according to Alef and Nannipieri [40]. Subsequently, to have unique conditions, soil samples were adjusted to 55% WHC. In order to test the temporal stability influenced by the adsorption and binding effects with increasing contact time in the soil (=aging), samples without natural DON and ZEA contents from Auweihler (sand), Ascheberg (clay), Berrenrath (silt), and Frankenforst (silty shell clay) (Table 1) were spiked with DON and ZEA and extracted immediately (0 days) and after 28 days. One part of the samples was stored in vessels with perforated aluminum foil at 8 °C, and the other part was stored at room temperature (21 °C) until extraction. To test aging with samples containing natural amounts of mycotoxins, soil samples from Lüttewitz and Rackwitz (Table 1) were also analyzed for DON and ZEA immediately after sampling (0 days) as well as 28 days after sampling after being stored in vessels with perforated aluminum foil at 8 °C as well as at room temperature (21 °C). The water content of the soil samples was controlled every five days and kept constant throughout the experimental period (28 days) via weight loss.

## Figures and Tables

**Table 1:** Optimized MS/MS parameters for the analyzed mycotoxins.

Mycotoxin	Retention Time (min)	Precursor Ion (m/z)	Product Ions (m/z)	Collision Energy (V)	Cone Voltage (V)
DON	2.92	297 [M + H]+	175.1/203.2	28/11	22
^13^C_15_-DON	2.92	312 [M + H]+	186.5	29	22
ZEA	7.40	319 [M + H]+	185.1/186.9	23/15	22
^13^C_18_-ZEA	7.40	337 [M + H]+	168.2	32	22

**Table 2:** Selected soil samples for method validation.

Sample	SOC (g/kg)	Clay (%)	Silt (%)	Sand (%)	Major Reference Soil Group
Ascheberg *	6.1	69	28	4	clay
Auweihler *	4.3	1	3	96	sand
Berrenrath *	18.9	16	75	7	silt loam (silt)
Frankenforst *	21.2	34	36	30	silty clay loam (mix)
Lüttewitz **	20.6	14	83	3	silt loam
Rackwitz **	20.5	14	53	33	silt loam

* without natural amounts of DON and ZEA ** with natural amounts of DON and ZEA.

**Table 3:** Apparent recovery (R*_A_*) and the relative standard derivation of R*_A_* (RSDr; n = 6) of spiked DON and ZEA in soils from Ascheberg, Auweihler, Berrenrath, and Frankenforst extracted with different methods and different extraction solvents from De Pereira et al. [39]; De Santis et al [24]; and Mortensen et al. [25,26]. The displayed recovery rate is the mean value of the recovery rate of all tested soil samples; ± the relative standard deviation.

	R*_A_* ± RSDr (%)			
	ACN *	H_2_O/ACN **	MeOH/H_2_O *	ACN/H_2_O/AcOH **
		(80:20)	(90:10)	(79:20:1)
**Ultrasonic bath for 1 h**				
DON	56 ± 21	68 ± 8	69 ± 4	85 ± 5
ZEA	51 ± 5	69 ± 9	67 ± 5	86 ± 4
**shaking for 24 h**				
DON	46 ± 21	65 ± 12	64 ± 8	69 ± 9
ZEA	42 ± 19	49 ± 5	65 ± 6	71± 5
**accelerated solvent extraction**				
**DON**	26 ± 10	30 ± 11	32 ± 9	35 ± 8
ZEA	31 ± 3	24 ± 6	42 ± 5	43 ± 6
**Pressurized solvent extraction**				
DON	27 ± 8	32 ± 9	36 ± 7	38 ± 8
ZEA	23 ± 6	34 ± 5	32 ± 8	43 ± 7

H_2_O = ultra-pure water; ACN = acetonitrile; MeOH = methanol; AcOH: acetic acid (glacial)* Mortensen et al. [25,26]. ** Pereira et al. [39]; De Santis et al. [24].

**Table 4:** Coefficient of determination (R^2^) and *p*-value (***) obtained for DON and ZEA in neat solvent mixture A/B (50:50) and spiked soil samples with different textures (Auweihler 96% sand; Ascheberg 69% clay, Berrenrath 75% silt; Frankenforst mixture). Soil samples were spiked before extraction.

Mycotoxin	Concentration Ranges	R^2^ and *p*-Value (***)				
		Neat Solvent Mixture A/B (50:50)	Sand	Clay	Silt	Mix
**DON**	1–100 ng/mL	0.9983 ***	0.9894 ***	0.9895 ***	0.9924 ***	0.9967 ***
**ZEA**	0.5–100 ng/mL	0.9996 ***	0.9944 ***	0.9886 ***	0.9928 ***	0.9926 ***

**Table 5:** LOQs, apparent recovery (R*_A_*), signal suppression/enhancement (SSE), extraction recovery (R*_E_*), and relative standard deviation (RSD_r_) of repeatability of R*_A_* for DON and ZEA in each validated soil texture.

Soil Texture		DON	ZEA
sand	LOQ (ng/g)	1	0.5
	R*_A_* (%)	86	83
	SSE (%)	88	82
	R*_E_* (%)	98	102
	RSD_r_ (%)	8	7
clay	LOQ (ng/g)	1	0.5
	R*_A_* (%)	87	85
	SSE (%)	90	89
	R*_E_* (%)	97	96
	RSD_r_ (%)	6	8
silt	LOQ (ng/g)	1	0.5
	R*_A_* (%)	85	82
	SSE (%)	86	83
	R*_E_* (%)	99	99
	RSD_r_ (%)	9	6
mix	LOQ (ng/g)	1	0.50
	R*_A_* (%)	83	82
	SSE (%)	83	84
	R*_E_* (%)	100	98
	RSD_r_ (%)	8	9

**Table 6:** Apparent recovery for DON and ZEA in spiked soil samples with different textures (Auweihler 96% sand; Ascheberg 69% clay; Berrenrath 75% silt; and Frankenforst mixture) after0 days and after 28 days stored at both 8 °C and 21 °C.

**Apparent Recovery after 0 days (%)**				
**Mycotoxin**	**sand**	**clay**	**silt**	**mix**
DON	85.65	87.28	84.77	83.19
ZEA	83.41	85.39	81.78	82.01
**Apparent Recovery after 28 days at 8 °C (%)**				
**Mycotoxin**	**sand**	**clay**	**silt**	**mix**
DON	84.56	87.78	85.57	82.67
ZEA	84.44	86.47	87.94	87.47
**Apparent Recovery after 28 days stored at 21 °C (%)**				
**Mycotoxin**	**sand**	**clay**	**silt**	**mix**
DON	85.12	87.23	84.89	83.34
ZEA	86.98	86.65	84.44	82.34

## Data Availability

Data is contained within the article.
